# Proactive Alleviation Procedure to Handle Black Hole Attack and Its Version

**DOI:** 10.1155/2015/715820

**Published:** 2015-10-01

**Authors:** M. Rajesh Babu, S. Moses Dian, Siva Chelladurai, Mathiyalagan Palaniappan

**Affiliations:** ^1^Department of CSE, Karpagam College of Engineering, Coimbatore 641032, India; ^2^Department of CSE, Shanmuganathan Engineering College, Pudukkottai District 622507, India; ^3^Department of IT, Nandha Engineering College, Erode 638052, India; ^4^Department of CSE, Sri Ramakrishna Engineering College, Coimbatore 641022, India

## Abstract

The world is moving towards a new realm of computing such as Internet of Things. The Internet of Things, however, envisions connecting almost all objects within the world to the Internet by recognizing them as smart objects. In doing so, the existing networks which include wired, wireless, and ad hoc networks should be utilized. Moreover, apart from other networks, the ad hoc network is full of security challenges. For instance, the MANET (mobile ad hoc network) is susceptible to various attacks in which the black hole attacks and its versions do serious damage to the entire MANET infrastructure. The severity of this attack increases, when the compromised MANET nodes work in cooperation with each other to make a cooperative black hole attack. Therefore this paper proposes an alleviation procedure which consists of timely mandate procedure, hole detection algorithm, and sensitive guard procedure to detect the maliciously behaving nodes. It has been observed that the proposed procedure is cost-effective and ensures QoS guarantee by assuring resource availability thus making the MANET appropriate for Internet of Things.

## 1. Introduction

Earlier communication technique was Human to Human (H2H) which gradually turned into Human to Machine (H2M), and now it took a wider leap into the Machine to Machine (M2M) communication called Internet of Things (IoT) [1]. IoT is the scenario, where all the devices in the network are no longer computers but “things.” Things would include cars, home automation sensors, surveillance cameras [2], containers in cargo, and trucks on transit. IoT paradigm proposes to connect all physical objects in a global Internet-based infrastructure to exchange information and communication. IoT aims to support intelligent identification, location, tracking, monitoring, and management. IoT is therefore based upon the integration of several communication solutions, identification and tracking technologies, sensor and actuator networks, and distributed smart object [3, 4]. IoT promises a smart environment that would offer immense savings of time, energy, and resources. For example, in real time world, the IoT scenario applied for smart homes appliances like refrigerators, air conditioner, and some other electronic goods is controlled by the mobile communication. This was the suitable example for the IoT scenario which will work based on the following concepts.

**Figure 1:**
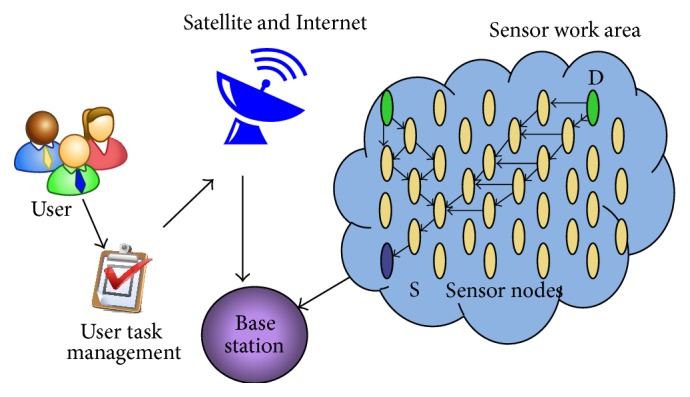
Wireless sensor network architecture.

Figure 1 declares the wireless sensor network architecture with regard to space and distributed autonomous sensors to monitor environmental conditions on the space, something just mentioned like pressure, sound, temperature, and so on. The WSN is built with many nodes; maybe it is varied from several hundred, where each node is connected to one sensor (or sometimes several sensors). All those nodes are working for sensors only. WSNs are an area monitoring application. WSN is deployed over a region, where any state is to be monitored.

IoT is the concept where the future environment will be surrounded by smart objects with interdependent networks of smart communication protocols such as Mobile Network Protocols of GSM and GPRS and IP Multimedia Subsystem Protocols and Communication Services, smart homes such as smart homes appliances like refrigerators, air conditioners, and water heaters, smart traffic such as GPS navigation rear-view mirror [2], smart city such as surveillance camera based observation of a person or group of places, and smart health such as patient online surveillance system and artificial human part artifact that has been created by someone or some process in medical domain. Enormous number of services will be made available to interact with these “smart objects” like sensor, and so forth. These objects are used in internet for sending a query, changing their state and conveying the information associated with them. While taking all this into account, security and privacy issues are going to be very huge. It thus creates a world where the physical objects are perfectly integrated into the information network and where the physical objects can become active participants in areas like business processes, life critical applications, and so forth.

Moreover, from the technical point of view, the basic idea behind this concept is the ubiquitous presence of a variety of things or objects such as radio-frequency identification (RFID) tags, sensors, actuators, and mobile phones. These objects are able to interact with each other and cooperate with their neighbors through unique addressing schemes like Session Management, Multimedia Services over IP Networks, Time Synchronization and Network Mobility [5]. World Wide Web (a merger of networks and databases) [6] was heavily used around the network users. IoT has more schematic domains; particularly wireless sensor networks [7] and ad hoc networks are two main preliminary domains. Therefore, the biggest challenge in this integral part of the future Internet is the caliber of utilizing the existing technology like wired and wireless communication. The realm of IoT cannot exist without the help of ad hoc network [4].

For instance, the objects should communicate with each other, whether an infrastructure exists or not. IoT increasingly involves a number of sensitive systems that will have to be protected immensely. Thus securing IoT especially sensor network is vital. However, the usage of the “smart objects” in wireless communication and ad hoc networks focus on the tremendous security challenges. These challenges alters the communication between two parties, which makes them unsecure. Attacking neighbor sensing protocols like SYN attack, UDP attack and authentication server attack spoils the transport layer session and provides hijacking [8] for unauthorized TCP session.

Among those attacks of packages data into IP datagrams, the network layer attacks like black hole attack, wormhole attack, rushing attack, sinkhole attack, and gray hole attack are more serious; particularly black hole attack in networking is more vulnerable whose underlying idea is to inject them in the active path from source to destination or to absorb the network traffic. As an experiment, the versions of black hole attack are massive threat to IoT, since it has the potential to distrust the entire network of IoT objects or part of the network, whose participation is essential to carry out the mission-critical and time-critical applications. Disrupting the IoT network [4] may result in serious consequences like loss of life, revenue, property, and so forth.

## 2. Related Works

Wireless sensor network (WSN) and ad hoc networks are prone to several serious security issues like network layer attacks. Thus wireless sensor network, its integration into IoT, and the various security challenges are discussed as follows.

### 2.1. Wireless Sensor Networks

A wireless sensor network is a collection of autonomous nodes distributed spatially over a network, where each node is connected to two or more locations for the purpose of transmitting and receiving data with respect to the sensors [9] concisely used for communicating any medium in wireless domain. The key functions of WSN are broadcast and multicast, routing, forwarding, and route maintenance. Each of such sensor network nodes has several hardware components like a radio transceiver with an internal antenna or connection to an external antenna, a microcontroller, a power source, usually a battery, and so forth. The topology of the WSN can vary from a simple star network to a complex wireless mesh network. Applications of WSN are wider, like immense use in military applications such as ocean surveillance systems, battle field surveillance, and attaching microsensors to weapons for stockpile surveillance [10]. The abovementioned applications joined together into a whole of wireless sensor networks that are divided into three categories [9], namely, monitoring objects which include monitoring both living and nonliving objects, monitoring space, and monitoring the interactions between objects and space.

The first category monitoring objects can be exemplified by structural monitoring. It is possible to detect the mechanical impact like breakages of the structure by acoustic emissions, sensing the vibration modes, responses to stimuli, and so forth, on the structure of buildings [11], bridges [12], and so forth. Environmental monitoring is the best example for monitoring space. In environments like forests [9], mountains [13], and glaciers [10], WSNs are deployed for the purpose of gathering environmental factors for long period of time. Also other environmental details such as temperature, humidity, moisture, and light sensor readings help in observing the environment. The combination of first and second category led to the third category monitoring interaction between objects and space. This comprises monitoring threats such as volcanic activities [14] and floods [15].

The last category deals with monitoring human beings. For example, in wearable computer jackets, the installed sensors would gather physiological information like heart beat rate, blood pressure, diagnosing patients with bipolar disorder [16], and also observing the patients in home care scenario [17]. Thus WSN applications have broadened its horizon in different fields. As WSN is one of the most important elements in the IoT, the benefits of connecting WSN and IoT [9] elements go beyond remote access, as heterogeneous information systems can be connected collaboratively to provide common services (new). It is important to consider the suitable approaches for the integration of WSN into the Internet. Wireless networks can be either infrastructure based networks or infrastructure less networks. In infrastructure based networks, communicating mobile devices are controlled and coordinated by base stations, whereas in infrastructure less networks, no centralized control point is present. Ad hoc networks fall under the category of infrastructure less networks. Security and privacy are the main issues in wireless networks [13]. If attackers exist, they can carry on a wide variety of attacks on the routing algorithm including selective forwarding, black hole, rushing, resource depletion, worm hole, and Denial of Service attacks.

Unfortunately, almost all wireless network routing algorithms are vulnerable to these attacks [13]. Moreover the various types of attacks are reduced based on many research articles; particularly Deng et al. studied the routing security issues and also analyzed black hole attack [12], where the problems are easily employed against the MANETs and they proposed solution for the same problem through AODV routing protocol. Thus we propose the new article for careful consideration to the black hole attack and reduce the same attacks on the WSN and ad hoc networks.

Panicker and Jisha proposed various attacks [8] in the MANET, particularly in network layer especially black hole attack, which is reduced by three mechanisms like TOGBAD, SAR protocol, and DPRAODV protocol [18] based reduction. It has some cons like protocol functionality, route distance, and network overload. To overcome this drawback, we introduced the new approach for hole detection algorithm to detect the maliciously behaving nodes and produce the cost-effective and ensure QoS guarantee by proactive alleviation procedure.

### 2.2. Ad Hoc Networks

In ad hoc networks, the nodes work in cooperation with each other in managing the network. In addition to acting as hosts, the nodes should also act as routers during the transmission. MANET is an ad hoc network, where the nodes are mobile in nature, cooperating with each other in network management, without the use of a network infrastructure or any centralized administration [13]. The primary goal of a MANET routing protocol is to establish a correct and efficient route between a pair of nodes, so that messages may be delivered in a timely manner.

Wu et al. proposed survey on attacks and countermeasures in MANET to achieve security goals, such as access control, authentication, availability, confidentiality, integrity, and nonrepudiation [19]. It also provides the MANET-IDS (Intrusion Detection Systems) to prevent attacks. Through this survey, we analyze the various ad hoc networks countermeasures in terms of packet delivery ratio and QoS guarantee such as network support, availability, and time consumption of the specific nodes on the networks.

Bhattacharyya et al. proposed DATA traffic attacks and CONTROL traffic attacks [20] to preserve the networks with respect to the RREQ and RREP methods. Thus network layer DATA traffic attacks are reduced by the proactive alleviation procedure.

Gagandeep and Kumar proposed [21] and discussed the various types of attacks under protocol stack and routing. Security issues associated with mobile ad hoc network attacks were classified based on the active and passive attacks. In particular, the active attacks like timing attacks are reduced based on the rushing attacks against on-demand routing protocols. These procedures are helpful to us to reduce the timing attacks like the ad hoc networks.

### 2.3. Integration of WSN and the Internet

**Figure 2:**
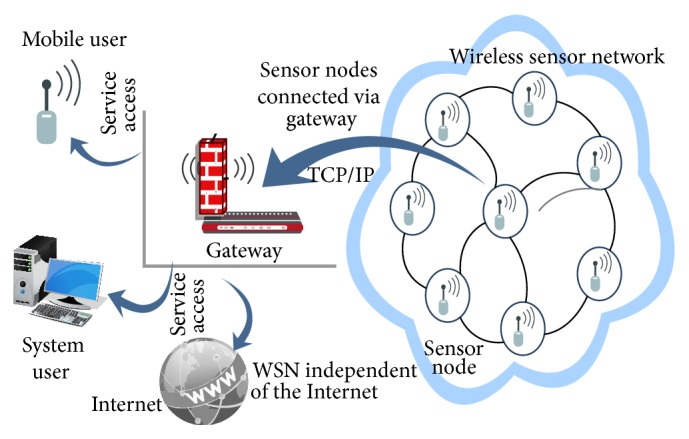
Independent networks.

Wide range of various sensor network applications, network embedded sensing or controlling devices were constructed by different databases, hardware platforms, and middleware and operating systems. To design the architecture of sensor, most application environments of sensor network are designed in tightly coupled closed architectures. From numerous advanced monitoring and control applications, wireless sensor networks [9] connect with the Internet by Tiny TCP/IP implementation, spatial IP address assignment, shared context header compression, application overlay networking, and distributed TCP caching. Thus we propose the following approaches for connecting the WSN to the Internet based on three methods: independent network, hybrid network, and access point network [22]. Since most of the WSNs access the Internet, both independent WSN and the internet can be connected through a single gateway. This falls under the first approach which is illustrated in Figure 2. A WSN can be completely independent of the Internet (front-end), be able to exchange information with Internet hosts (gateway), or share a compatible network layer protocol (TCP/IP).

**Figure 3:**
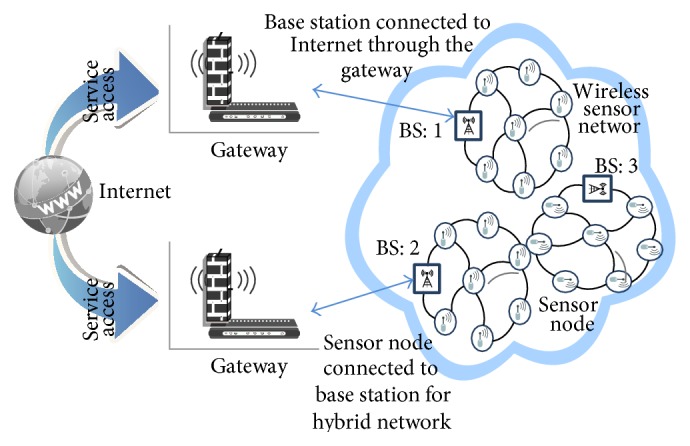
Hybrid networks.

As there is a dramatic increase in the integration, the second approach has come into existence. In Figure 3, the level of integration of both the independent networks depends on the actual location of the nodes, which provides access to the Internet. These nodes can be a few dual sensor nodes (e.g., base stations) located on the root of the WSN (hybrid).

**Figure 4:**
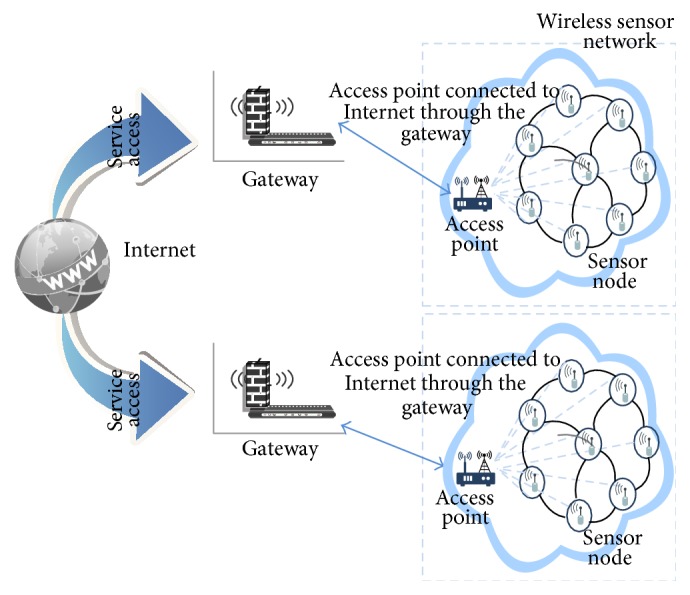
Access point networks.

Figure 4 illustrates the third approach, which is the current WLAN structure that forms an access point network. This network allows the sensing nodes to access the Internet in one hop (access point).

The access point approach is also called the TCP/IP solution. The sensor nodes implement the TCP/IP stack; thus they can be considered as the complete backbone of the Internet. Any Internet host can open a direct connection with them, and vice versa. Moreover, this solution fully integrates the WSN with the IoT [9].

### 2.4. Challenges in Integrating WSN and the Internet

The potential tasks to be accomplished by the sensor nodes are network configuration, Quality of Service, and security [23].

#### 2.4.1. Network Configuration

Network configuration based on wireless medium is processed by the identification of premises and correct wireless driver availability and also configuration of interface on the sensor network. Afterwards choose managing wireless connections wireless management tools. Particularly sensor nodes are also required to control the WSN configuration, which covers various tasks, like ensuring scalable network constructions, ensuring self-healing capabilities by detecting and eliminating faulty nodes, and also managing their own configuration. However in order to achieve better caliber of the network and its operations, means of network configuration and management is necessary.

#### 2.4.2. Quality of Service

The Quality of Service which includes the average delay, lifetime of node, throughput, switching delay, and mobile and multiple sinks is of greater importance to WSN topology. To improve service management in terms of IoT, the gateways act only as repeaters and protocol translators. Sensor nodes should ensure the Quality of Service through optimum utilization of resources of all the devices that take part in IoT. With respect to balancing of workload between the nodes that offer available resources, it is necessary to improve the QoS for mechanisms that require high amount of resources like security mechanisms. Since rapid changes in the link characteristics of the nodes can lead to considerable reconfiguration of the WSN topology, it is very essential to find new approaches to ensure service guarantees.

#### 2.4.3. Security

Commonly in WSNs, the sensor nodes play an important role in ensuring data confidentiality, integrity, availability, and authentication depending on the sensitivity of the application. However, the scenarios for an attack need to present physically almost near the targeted WSN, in order to collapse the network by introducing malicious nodes. Thus when WSNs openly access the Internet, such nearness in the location will no more be required and attackers would be able to threaten WSNs from everywhere. Therefore, novel security mechanisms must be developed with the resource constraints in concern to protect WSNs from the attacks originating from the Internet.

### 2.5. Security Challenges

While WSNs are the essential part of the IoT, several security challenges are also under consideration. It is essential to consider the security of the IoT from a global perspective rather than an issue that is related to a particular technology. As discussed previously, TCP/IP solution is the best solution to effectively integrate WSN and the Internet. However, there are multiple factors to be considered, in order to choose the suitable integration approach [new]. The main factors are summarized below.

#### 2.5.1. Resilience

Any WSN that directly provides its services to external entities is quite vulnerable to attacks. For example, it is very easy to launch a Denial of Service (DoS) attack due to the throughput of the transmission medium and the capabilities of the sensor nodes. Thus gateways and sensor nodes must comprise security mechanisms against such attacks.

#### 2.5.2. User Authentication and Authorization

Every node in a network might not be scalable for long-lived applications; thus it is necessary to consider the implementation of single sign-on systems like Kerberos to authenticate and authorize the user [17].

#### 2.5.3. Security of the Communication Channel

It is considered that IPsec might be too “heavy” for constrained WSN [22]. Therefore, it is necessary to analyze other mechanisms such as TLS to offer an end-to-end secure channel. In fact, it is also necessary to study the different key exchange mechanisms that should be used in this context.

#### 2.5.4. Accountability

For an Internet-enabled WSN, it is essential to develop a distributed system that is able to record the interactions with the users of the system. By storing all interactions, we are able to recreate security incidents and abnormal situations.

#### 2.5.5. Functionality

In some applications, the sensor nodes do not need to be aware of the Internet. For example, while the tasks of WSN are limited to collecting information and answering users' queries, it is not necessary to contact any Internet service.

#### 2.5.6. Hardware

A specially constrained sensor node might not be able to directly connect to the Internet due to the memory requirements of the different security mechanisms (e.g., AES-128, Elliptic Curve Cryptography) and the Internet protocols and standards (e.g., HTTP, web services).

#### 2.5.7. Inherent Weaknesses

Internet-enabled sensor nodes are vulnerable to many different types of attacks, ranging from DoS attacks to exploit attacks. This particular factor is actually quite important in choosing whether certain applications should completely isolate their sensor nodes from the Internet, filtering all traffic at the edge of the network.

#### 2.5.8. Network Redundancy

A group of sensor nodes may offer the same functionality for redundancy purposes. But in a TCP/IP environment, an external host will request services from specific nodes through their IP addresses. Thus it is necessary to develop specific mechanisms in TCP/IP environments to deal with exceptional circumstances (e.g., unreachable nodes).

#### 2.5.9. Protocol Optimization

Most WSN-specific protocols include certain mechanisms that allow a network to self-heal and to optimize its internal behavior.

## 3. Attacks in WSN and Ad Hoc Networks

### 3.1. Wormhole Attack

There are two unauthorized parities which interrupt and communicate on the network which are called wormhole attack. From wormhole attack, an attacker receives packets at one point in the network, “tunnels” them to another point in the network, and then replays them into the network from that point [24]. Routing can be disrupted, when routing control messages are tunneled. This tunnel between two colluding attackers is referred to as a wormhole. Packet leashes are used to combat wormhole attacks.

### 3.2. Sinkhole Attack

Sinkhole is a more complex attack [22] compared with black hole attack. Given certain knowledge of the routing protocol in use, the attacker tries to attract the traffic from a particular region through it. Other nodes will then consider the path through this attacker node better than the currently used one and move their traffic onto it. Since affected nodes depend on the attacker for their communication, the sinkhole attack can make other attacks like gray hole, black hole, and so forth.

### 3.3. Rushing Attack

Two colluded attackers use the tunnel procedure to form a wormhole. If a fast transmission exists between the two ends of the wormhole, the tunneled packets can propagate faster than those through a normal multihop route. This is called the rushing attack [17]. The rushing attack can act as an effective DoS attack.

### 3.4. Gray Hole Attack

Gray hole attack has two phases. In the first phase, a malicious node exploits the AODV protocol to advertise itself as having a valid route to a destination node, with the intention of intercepting packets, even though the route is spurious. In the second phase, the node drops the intercepted packets with a certain probability. In this gray hole attack, routing misbehavior leads to dropping of messages packets selectively. These will be rectified by secure signature algorithm to trace the packet dropping nodes on ad hoc network. Gray hole attack is a variation of black hole attack in which the normal node will suddenly behave maliciously and start dropping packets selectively. Thus detecting this kind of attack is more challenging [25].

### 3.5. Black Hole Attack

Clearly MANET and WSN are vulnerable to various attacks. Attacks in the network layer have two purposes: not forwarding the packets or modifying some parameters of routing messages. A basic attack that an adversary can execute is to stop forwarding the data packets. A black hole attack is a kind of Denial of Service, where a malicious node sends a forge reply to the source node that it possesses a shortest route to the destination. The source will establish a connection by forwarding packets to the adversary. The adversary in turn will discard those packets without forwarding them to the destination. Cooperative black hole attack is an attack where multiple black hole nodes perform in coordination.

In [11], it is emphasized that, in order to ensure the security between two communicating nodes, a protocol must enable the destination node to identify the source of a given message, and the source node must be able to authenticate the legal destination node. The work done in [15] presents a reputation-based routing scheme for hierarchical ad hoc networks and the cluster head, acting as the reputation manager for updating reputation information. Thus, the malicious nodes would be isolated for safeguard routing security. In [14], a secure routing protocol with payment mechanism that prevents node selfishness for mobile ad hoc networks is proposed. A source node can securely send a confidential message to the destination node through a number of intermediate nodes. In addition, all messages transmitted between nodes should be verified and protected in the protocol.

In [26], anti-black hole mechanism is discussed. Every node is subjected to an estimation of the suspicious value. The suspicious value is found based on the amount of abnormality in RREQ and RREP packets of the node. When the suspicious value exceeds a threshold value, the node is identified as a black hole and the Intrusion Detection System (IDS) will blacklist the node and the time of identification. Thus the cooperative black hole nodes can be identified. The drawback is that the mobile nodes have to maintain training data and regular updates.

In [27], CBDAODV mechanism is proposed. A source node will accept at least two RREP packets from different replying nodes. Thus by utilizing another routing path, the source node itself can evaluate the reliability of the currently selected route and make a rerouting decision once it suspects the reliability of currently selected route. Through another route, a confirmation control packet which consists of the name of the second malicious node to which the first malicious node sends the data packets is sent. On receiving the packet, the destination node will reply to indicate the existence of the route between the destination and the malicious node. If the reply packet indicates that no path exists, the source node now switches its routing path to the alternate route and retransmits its data packets. Also the malicious nodes are put to observation, to identify whether the nodes regularly work in cooperation with each other.

In [28], a solution is proposed by modifying the AODV protocol to avoid multiple black holes in the group. It maintains a fidelity table. Every participating node is given a fidelity level that tells the reliability of that node. Any node having value as 0 is considered as malicious node and is eliminated from the network. The fidelity levels of the nodes along a route are increased on every successful transmission of the data; otherwise the fidelity level of the nodes is decreased. The processing delay in the network is high.

In [29], MOSAODV mechanism is presented, where a timer is set in the source node to collect all the RREP packets and those packets with exponentially high destination sequence number are discarded. In [29], all the RREPs are stored in the newly created table, until the modified wait timer. The modified wait timer is initialized to be half the value of RREP wait time, which is the time for which source node waits for RREP control messages before regenerating RREQ. The source node after receiving first RREP control message waits for modified wait time. For this time, the source node will save all the coming RREP control messages in the new table. Subsequently, the source node analyses all the stored RREPs from the new table and discards the RREP having very high destination sequence number. The node that sent this RREP is suspected to be the malicious node. Once such malicious node is identified, it can discard any control messages coming from that node. Now since malicious node is identified, the routing table for that node is not maintained.

In [30], DPRAODV mechanism is proposed to encounter black hole attacks in AODV. In normal AODV, the node that receives the RREP packet first checks the value of sequence number in its routing table. The RREP packet is accepted, if it has RREP_seq_no higher than the one in routing table. In the proposed mechanism, an additional check is performed to find whether the RREP_seq_no is higher than the threshold value or not. The threshold value is dynamically updated as in [13] in every time interval. As the value of RREP_seq_no is found to be higher than the threshold value, the node is suspected to be malicious and it adds the node to the blacklist. As the node detects an anomaly, it sends a new control packet ALARM to its neighbors. The ALARM packet has the blacklist node as a parameter, so that the neighboring nodes know that RREP packet from the node is to be discarded. Thus the malicious node is isolated from the network by the ALARM packet. The threshold value is the average of the difference of dest_seq_no in each time slot between the sequence number in the routing table and the RREP packet. In this mechanism, the threshold of a valid RREP sequence number is derived dynamically to evaluate the sequence numbers of the received RREP packets for every route request.

In [18], a mechanism is proposed to defend cooperative black hole attack. Each node observes the data forwarding nature of its neighboring node. This information is recorded in a DRI (Data Routing Information) table. Each node maintains an additional DRI table. In the DRI table, 1 stands for “true” and 0 for “false.” The first bit stands for information on routing data packet from the node, while the second bit stands for information on routing data packet through the node. If the entry is 0 for a node N implies that a node has not routed any data packets from or through N. An additional cross-checking method is also done. The source node broadcasts a RREQ message to discover a secure route to the destination node. The intermediate node (IN) generating the RREP has to provide its next hop node (NHN) and its DRI entry for the NHN. Upon receiving RREP message from IN, the source node will check its own DRI table to see whether IN is a reliable node or not.

## 4. Proposed Proactive Black Hole Alleviation System

The proposed black hole alleviation system adopts a mechanism that would proactively detect the black hole nodes and isolates them in order to ensure a secure communication.

The various notations used in the proposed system are tabulated along with the description as follows:

 
*a*_1_: source node,
 
*a*_*n*_: destination node,
 
*a*_*i*_: intermediate node, where *i* = 2,3,…, *n* − 1,
 {*p*_1_, *p*_2_,…, *p*_*n*_}: possible nodes between *a*_*i*_, and *a*_*i*+1_
 
*g*_*i*_: geographical region, where *i* = 1,2,…, *n*,
 
*t*: time in minutes,
 {*D*_1_, *D*_2_,…, *D*_*n*_}: monitoring units for regions {*g*_1_, *g*_2_,…, *g*_*n*_},
 
*s*: time in minutes or seconds.

During transmission, the data originated from the source node is forwarded by the intermediate nodes and finally reaches the destination. However from one node to another node, there should exist 1 to *n* paths in the direction towards the destination. If no path exists, it means there are no objects, or it may be the result of black hole attack.

From source *a*_1_ to destination *a*_2_, there exists the increment of *a*_*i*_ nodes. For *a*_1_ to *a*_2_, there may exist *p*_1_ to *p*_*n*_ (possible nodes in the path to become *a*_2_). After fixing up *a*_2_, for *a*_2_ to *a*_3_ there may be *p*_1_ to *p*_*n*_. At every node, there exist *p*_1_ to *p*_*n*_ alternatives. Therefore any *a*_*i*_ in the path from *a*_1_ to *a*_*n*_ is the outcome of {*p*_1_, *p*_2_,…, *p*_*n*_}.

Moreover, each and every node in the path *a*_1_ to *a*_*n*_ is geographically distanced. Therefore in this task, the nodes from the same geographical region for every set {*p*_1_, *p*_2_,…, *p*_*n*_} are considered as a cluster *g*_*i*_. The nodes form closed knowledge sharing networks at various geographical levels which gives a structure to identify the black hole like attacks:

 For (*i* = 1; *a*_*i*_ ≤ *a*_*n*_; *i* + 1)
 if (*n* > 3)  ∖∖ where “*n*” is the number of possible nodes
 there exist a region *g*_*i*_ = {*p*_1_, *p*_2_,…, *p*_*n*_}
 else if (*n* < 3)
 there does not exist a region *g*_*i*_
 trigger Hole detection Procedure
 while (*n* ≥ 1).

Therefore, single active node present in that region {*p*_1_, *p*_2_,…, *p*_*n*_} is enough to uphold the route path through that region. If *a*_1_ is in *g*_1_ (similarly *a*_*n*_ is in *g*_*n*_ ), then the geographical region is constituted at least with the minimum of three nodes. If there does not exist a geographical region, then a single active node in that region {*p*_1_, *p*_2_,…, *p*_*n*_} can uphold the route path through that region. The challenge is to keep at least one node in the geographical region active to accomplish the mission. However the attacks like worm hole, black hole, and so forth have the potential to completely disrupt the network of nodes in a single geographical region or more, thus leaving the network unoperational. Therefore to detect the black hole attack, two methods are proposed here. If the path between *a*_1_ and *a*_*n*_ is mission critical, then the following algorithm should be implemented. The following procedures are useful to make the proposed methodology concise against the black hole data centric attack in MANET and wireless sensor network.

### 4.1. Timely Mandate Procedure

Timely updating of the node status is done from the monitoring units based on the availability requirements. Set of {*g*_1_, *g*_2_,…, *g*_*n*_} may be connected to single or more monitoring units. Monitoring units may be data collecting point or nearby server, and so forth. One is timely updating of the node status to the monitoring units. This monitoring is designed based on the requirement for a specific application to take place. For instance, a health of the person is monitored from the remote place by the doctor; in that case he may collect 1 sample a day or 2 or more samples a day based on the health condition of the patient. The time is scheduled by the doctor. Consider {*g*_1_, *g*_2_,…, *g*_*n*_} connects *a*_1_ to *a*_*n*_. If data is collected at every *t*, then nodes in the region {*g*_1_, *g*_2_,…, *g*_*n*_} are scheduled to respond to {*D*_1_, *D*_2_,…, *D*_*n*_} in (*t* − *s*) seconds earlier, respectively. At any *D*_*i*_, if the status of any *a*_*i*_ in any *g*_*i*_ is not updated before (*t* − *s*) seconds, then the node is considered unavailable.

### 4.2. Hole Detection Procedure

This algorithm is triggered based on untimely reply, on element of doubt, or on special cases, and so forth. If a black hole intends to steal only the data packets, then it will not obstruct the routing process; this includes path discovery. If the black hole does not obstruct the routing, then the existing techniques can be fooled. Therefore, the hole detection algorithm transmits the data packets and carefully records the status of the acknowledgement. However, the hole detection algorithm randomly generates the message and broadcasts the message to the next hop.

The first few data bits are considered as flags. The flags are as follows:

 
*Resource (R)*: if the packet is sent as a message this flag is set to “1.”
 
*Warning (W)*: if this flag is set to “1” then it serves as warnings about certain nodes being a black hole component.
 
*Acknowledgment (A)*: if this flag is set to “1” the received packet is an acknowledgement.
 
*Power (P)*: the status of power is mentioned.
 
*Unique nonce (N)*: number used only once in the communication and generated by a separate formula by each and every node, where *i* is the serial number of the node {1,2,…, *n*}.
 
*Unique ID (I)*: since no standard naming convention exists for sensor nodes this 16-bit number is unique to each and every sensor.
 
*Geographical serial number (G)*: this number contains two octets and is written in the form (*g* · *n*), where *g* is the region number and *n* is the serial number of that node.

In the data packet, the following details are placed {R‖P‖N‖I‖G}: if R is present then it is an enquiry, and it should be acknowledged. The remaining portion of the data packet is dumped with the random messages and then broadcasted to the next hop. Those who receive the enquiry packet after verifying it will acknowledge the sender with {A‖P‖N‖I‖G}. In case, if a node is compromised, it will not send the reply or send the irrelevant reply. Based on that the sender after being timed out by the next hop or through getting the irrelevant reply warns the other nodes in the region *g*_*i*_ with the notification message {A‖N_S_‖I_S_‖G‖I_*i*_} called black hole intimation packet, where the suffix marks the source node. Suffix “*i*” marks the ID of the suspected node as being black hole.

### 4.3. Sensitivity Guard Procedure

The sensitive geographical region is one where the possibility of attack is high. For instance, the nodes deployed to monitor the movements of enemies [12], and so forth. To monitor the attack, complex security procedures as proposed for path discovery and path reversal can be used [24]. Moreover the public key algorithm RSA is used, since it gives better performance over other public key encryption schemes [16]. Since the sender of the data needs to be authenticated, RSA digital signature scheme is applied to the enquiry packet with {R‖P‖N‖I‖G}, the acknowledgement packet {A‖P‖N‖I‖G}, and the black hole intimation packet {A‖N_S_‖I_S_‖G‖I_*i*_}. However before using the public key certification, the data packet is padded with OAEP. After the padding is done, the message is encrypted with the private key of the sender which will then be decrypted by the receiver with the public key of the sender.

## 5. Result Analysis

Many datasets relevant to wireless sensor networks are collected from various providers like I-LENSE software and datasets, and so forth. After collecting the dataset, the attack conditions for black hole, wormhole, and so forth are applied in the dataset. Various algorithms are analyzed by applying the methods to the generated dataset. The result is presented in Table 1.

The analysis shows the irregular vulnerabilities that the existing methods have towards the black hole attack or to the version of black hole attack. This is an important analysis because it clearly proves that even the secure methods can be victimized by the version of black hole attack. Therefore, in the future if the ad hoc network is used for IoT, it will exhibit weakness towards this type of attacks. Hence it becomes highly risky for the mission-critical applications to function on the IoT. Various countermeasures are analyzed against the attacks and the result is presented in Table 2.

However, interestingly there are countermeasures, which have the potential to detect and mitigate one or few attacks but not all types of attacks. Moreover, many countermeasures are of reactive [31] type. This causes a slump in performance for some time period. This results in packet loss and delay in packet transmission, which is not tolerable in mission-critical applications.

**Figure 5:**
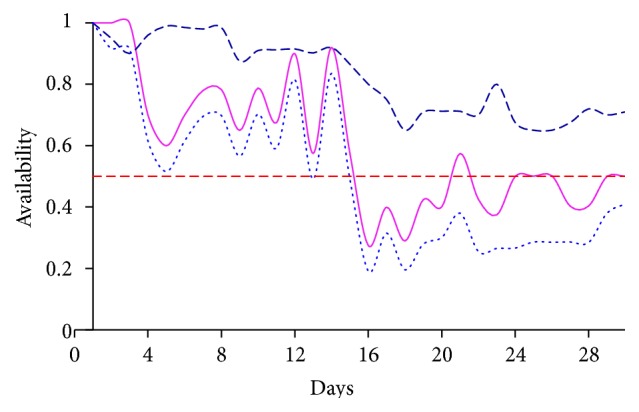
Measure of availability on dataset 1 with and without various detection procedures, where dotted blue line marks the detection with 2 methods and magenta line marks the detection with 3 methods, where dark blue line marks the detection with alleviation procedure.

**Figure 6:**
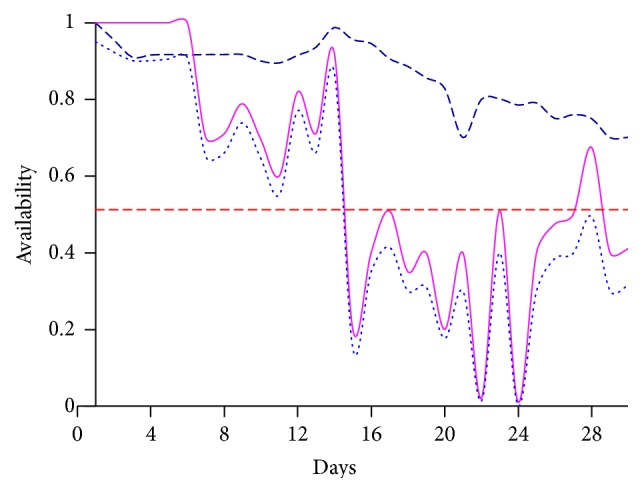
Measure of availability on dataset 2 with and without various detection procedures, where dotted blue line marks the detection with 2 methods and magenta line marks the detection with 3 methods, where dark blue line marks the detection with alleviation procedure.

According to Figures 5 and 6, it is evident that supporting the WSN infrastructure with necessary components rather than just implementing more countermeasures and security features yields better result. It is because those detection methods are costly and they consume a considerable amount of time to recover the system from the attack, which in turn will impact on the system performance. It makes the environment futile for mission-critical applications.

**Figure 7:**
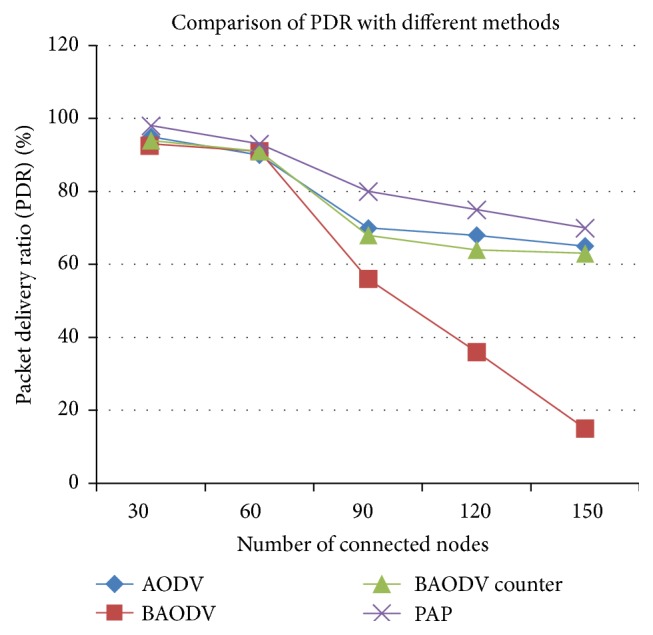
Measure of packet delivery ratio on dataset 3 with no attack, with black hole attack, with other countermeasures, and with the proactive alleviation procedure.

**Figure 8:**
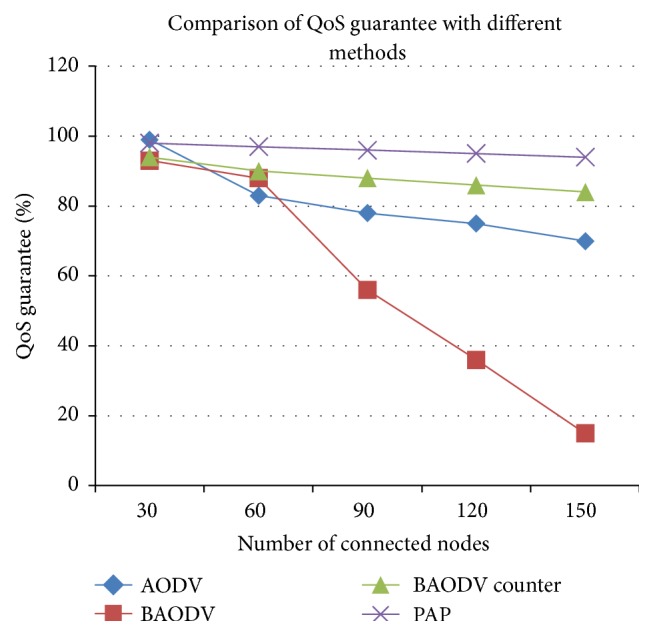
Measure of QoS guarantee on dataset 3 with no attack, with black hole attack, with other countermeasures, and with the proactive alleviation procedure.

Figures 7 and 8 show the results obtained based on experimentation on dataset 3. From Figure 7, a considerable amount of drop in percentage of packet delivery could be found, when there are multiple black hole attacks. The attacks are encountered with the countermeasures; however the countermeasures are not instantaneous. Thus there is a potential drop in the network performance as soon as attack is introduced. But time-critical applications cannot stand this nature. Thus the proposed alleviation procedure proves its proactive behavior that prevents the delayed detection of the attack, ultimately thwarting the drop in the packet delivery.

**Figure 9:**
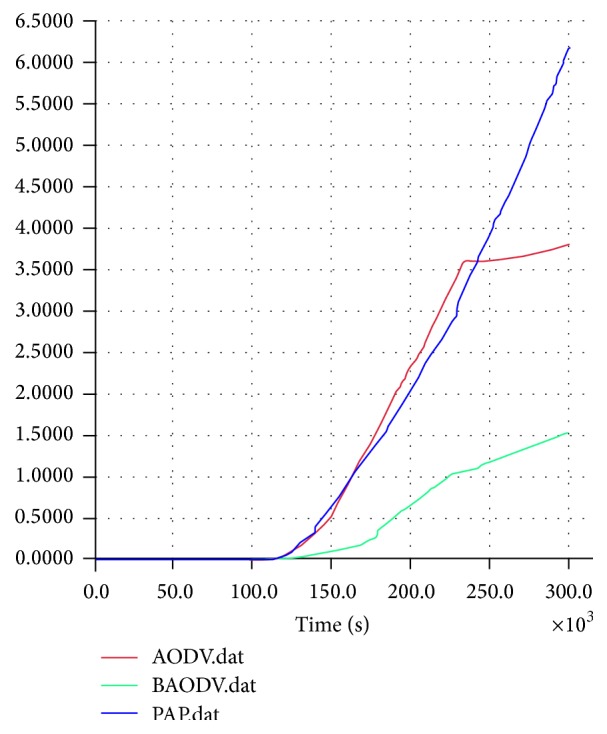
Measure of throughput on dataset 3 with no attack, with black hole attack, and with proactive alleviation procedure.

The Quality of Service guarantee of the network is analyzed in Figure 8. The network that adopts the alleviation procedure provides at least a minimum of QoS guarantee that keeps the network working, where the launch of an attack is immaterial. The throughput analysis is shown in Figure 9. The throughput significantly increases with respect to time, without any descent at any point in the network. Thus the timely monitoring of the environment and categorizing the environment based on regions and identifying sensitive regions, as well as ensuring security for critical regions more importantly ensuring minimum provisions, made the alleviation procedure unique and good enough to support the mission-critical applications.

## 6. Conclusion

IoT is used for future Internet user. But if the Internet user uses IoT effectively, there are many sets of facts that arise on the IoT. Consequently, the existing infrastructure which includes even the resource and power constrained WSN and ad hoc networks should adapt to the needs of the IoT to support various types of services. When we reduce those constraints like unauthorized data and control centric attack, IP configurations loss, and the environmental compatibility, the user faces number of various restrictions. From those restrictions, the most annoying thing is the data centric attack named black hole attack and its version. To solve this issue, we propose an architecture level solution to sustain the network to support the array of applications. To solve this issue, we propose an architecture level solution to sustain the network to support the array of applications. Thus the results of proactive alleviation procedure to handle black hole attack and its version's proposed algorithm prove better solution for black hole attack and its upcoming versions.

In the developing era of ad hoc networking and wireless sensor networks, many updated technologies are available. There are many technologies like microelectromechanical systems technology, digital electronics, and potential sensor networks applications which are applied for the IoT. In addition to that our proposed method applies many domain applications like environmental/earth sensing, industrial monitoring, entertainment industry, and so on.

## Figures and Tables

**Table 1:** Vulnerability analysis of routing protocols.

Attacks	Countermeasure	Effectiveness
Black hole	SAR	Effective
Wormhole	Packet leashes	Effective
Rushing attack	RAP	Versatile
Resource Depletion	IDS	Effective
Gray hole	Checkpoint based multihop ACK	Versatile
Sinkhole	Intruder detection	Versatile
DoS	IPS/IDS	Versatile

Effective: avoids or allows negligible damage.

Versatile: allows attack only to do a partial damage.

Ineffective: allows the attack to meet out considerable or full damage.

**Table 2:** Effectiveness of attack countermeasures.

Attacks	AODV	DSR	SAODV	ARAN	ARIADNE	SEAD	SAR
Black hole	V	PV	V	V	V	V	NV
Wormhole	V	PV	V	V	V	V	V
Rushing attack	V	V	NV	NV	NV	NV	V
Resource depletion	V	V	V	V	V	NV	NV
Gray hole	V	V	PV	PV	NV	V	NV
Sinkhole	V	V	V	V	V	V	V
DoS	V	V	V	V	NV	NV	V

V: vulnerable.

PV: partially vulnerable.

NV: not vulnerable.
